# Axillary Lymph Node Involvement in Breast Cancer: A Random Walk Model of Tumor Burden

**DOI:** 10.7759/cureus.6249

**Published:** 2019-11-27

**Authors:** Vincent Vinh-Hung, Nicolas Leduc, Jacqueline Baudin, Guy Storme, Nam P Nguyen, Clarisse Joachim, Elsa Cecilia-Joseph, Claire Verschraegen

**Affiliations:** 1 Radiation Oncology, University Hospital of Martinique, Fort-de-France, MTQ; 2 Engineering, University Hospital of Martinique, Fort-de-France, MTQ; 3 Radiation Oncology, Universitair Ziekenhuis Brussel, Brussels, BEL; 4 Radiation Oncology, Howard University, Washington DC, USA; 5 Epidemiology and Public Health, Cancer Registry, University Hospital of Martinique, Fort-de-France, MTQ; 6 Biostatistics, University of the French West Indies, Schoelcher, MTQ; 7 Oncology, Comprehensive Cancer Center, Ohio State University, Columbus, USA

**Keywords:** breast neoplasms, prognostic factors, lymph node ratio, auto-correlation, random walk, drift, recursive model

## Abstract

We reinvestigate the relationship between axillary lymph node involvement in breast cancer and the overall risk of death. Patients were women from the Surveillance, Epidemiology, and End Results (SEER) program, aged between 50 and 65 years, presenting a first primary T1-T2 (tumor size ≤5 cm), node-positive, non-metastasized unilateral breast carcinoma, diagnosed from 1988 to 1997, treated with mastectomy without radiotherapy. Hazard ratios (HRs) were computed at each percentage of involved nodes using the proportional hazards model, adjusting for the patient's demographic and tumor characteristics. The pattern of the hazard ratios was examined using serial correlations. Significance testing used the "portmanteau" test. Based on 4,387 records available for analysis, the relation between adjusted mortality and axillary lymph node involvement was modeled as H_t_ - H_t-__1_ = μ + a_t_, where t is the percentage of involved nodes, H_t_ is the mortality hazard ratio at the percentage t, μ is a constant, and a_t_ is white noise. The constant μ was estimated at 0.020, corresponding to a 2% increment in the mortality hazard ratio per 1% increase in the percentage of positive nodes. The model was considered acceptable by the "portmanteau" test (P=0.205). We conclude that the effect of the tumor burden might be expressed as a random walk difference model, relating the mortality hazard ratio with the percentage of involved nodes. We will use the model to explore how treatments affect the course of the disease.

## Introduction

In breast cancer patients undergoing axillary lymph node dissection, a variable number of lymph nodes are removed and examined. Some of these might be found uninvolved (i.e. negative nodes), while others are found involved by metastatic cells (i.e. positive nodes). The number of involved nodes is among the most powerful prognostic factors. It is used in cancer staging systems to classify cases of breast cancer into different prognostic groups, e.g. 1-3, 4-9, and 10 or more involved nodes correspond to the respective nodal stages N1, N2, and N3. We have, however, argued that the number of involved nodes is confounded by the number of nodes examined since the latter number might be affected by the extent of the surgical technique, by the normal anatomical variability between patients, and by the variability of the pathological examination [1-2]. For these reasons, a staging system based on the number of involved nodes might be less robust than a staging system based on the ratio or the percentage of involved nodes [3-4]. In an earlier investigation using the Surveillance, Epidemiology, and End Results (SEER) data, we noted that there was no clear change point in the relationship between the number of involved nodes and mortality [5]. We overlooked then the utility of the ratio. In the present paper, we revisit the older original data to evaluate whether a new perspective can be gained by analyzing the relationship between the lymph node ratio, expressed as the percentage of involved nodes (% positive lymph nodes among the lymph nodes examined), and the risk of death.

## Materials and methods

The selection of patients is the same as previously reported [5]: records of women presenting a first primary T1-2 (tumor size <= 50 mm) node-positive (at least one lymph node involved) non-metastasized unilateral breast carcinoma, diagnosed between 1988 and 1997, were abstracted from the SEER 9 registries [5]. The selection was limited to mastectomy without radiotherapy to avoid treatment interaction. Age was limited to >50 and <65 years to reduce the influence of age-related co-morbidity and to reduce the variability with age in the receipt of treatments [6-7]. After cleaning the data of discrepancies, e.g. more involved nodes than examined, 4387 records were available for analysis. The median follow-up for patients alive at cutoff date December 31, 2001, was 106 months (range 1-167).

Hazard ratios for overall survival were computed for each percentage of involved nodes cutoff (we will later label these as *H_t_*), adjusted by proportional hazard models that included indicators for geographical area, year of diagnosis, race, marital status, age, tumor histology, grade, hormone receptor status, and tumor location and size (Table 1). Considering that the successive *H_t_* hazard ratios are not independent observations, we modeled the Ht hazard ratios as ordered observations to represent the relation between mortality and the percentage of involved nodes. The adequacy of the final model was assessed using serial correlations (or auto-correlations). Significance testing that the autocorrelations differ from 0 used the "portmanteau" test [8].

**Table 1:** Patients characteristics and overall mortality hazard ratios Patients characteristics and overall mortality hazard ratios computed in a proportional hazards model that included the listed variables. The reference levels for the categorical variables (/) or the units for the continuous variables (U) are noted in square brackets. The numbers of nodes were replaced with the percentage of involved nodes in subsequent analyses. NA: not applicable

Characteristic	N	% of total	Hazard ratio for overall mortality (95% confidence interval)
Registry area			
East states	1,828	41.7%	1 (reference)
Central states (/East)	1,360	31.0%	0.92 (0.81–1.05)
Western states (/East)	1,199	27.3%	0.87 (0.77–0.98)
Year of diagnosis (continuous U: 1 year)			0.94 (0.92–0.96)
1988-1992	2,507	57.1%	NA
1993-1997	1,880	42.9%	NA
Race black (/non-black)	370	8.4%	1.42 (1.20–1.67)
Married status (/not-married)	2,955	67.4%	0.82 (0.74–0.91)
Age at diagnosis (continuous U: 1 year)			1.01 (1.00–1.03)
51-54	1,177	26.8%	NA
55-59	1,542	35.1%	NA
60-64	1,668	38.0%	NA
Histology ductal (/non-ductal)	3,468	79.1%	1.02 (0.90–1.16)
Pathological grade 3-4 (/other)	1,570	35.8%	1.36 (1.22–1.51)
Hormone receptor status, from 1990			
Estrogen negative (/non-neg)	713	16.3%	1.38 (1.16–1.63)
Progesterone negative (/non-neg)	999	22.8%	1.39 (1.19–1.62)
Tumor medial location (/non-medial)	476	10.9%	1.12 (0.96–1.31)
Tumor size (continuous U: 1 mm)			1.02 (1.02–1.03)
T1	2,081	47.4%	NA
T2	2,306	52.6%	NA
Number of nodes examined (continuous U: 1 node)			0.97 (0.97–0.98)
1-4	37	0.8%	NA
5-9	543	12.4%	NA
10-14	1,327	30.2%	NA
15+	2,480	56.5%	NA
Number of involved nodes (continuous U: 1 node)			1.08 (1.07–1.09)
1	1,561	35.6%	NA
2-3	1,428	32.6%	NA
4-9	967	22.0%	NA
10+	431	9.8%	NA

## Results

**Figure 1:**
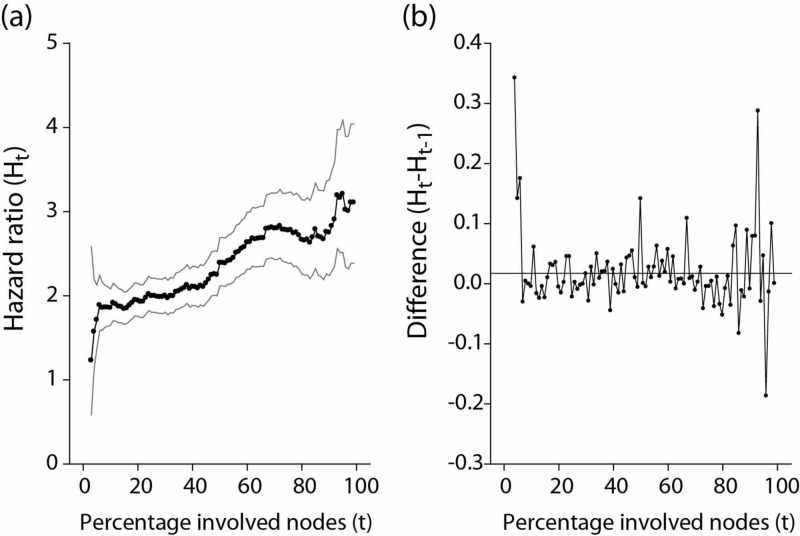
Mortality hazard ratios by percentage of involved nodes (a) Hazard ratios of any-cause mortality comparing higher percentages with lower percentages of involved nodes (vertical axis, *H_t_*), as a function of the percentage cutoff (horizontal axis, *t*). All hazard ratios were adjusted with other covariates (geographical area, age, race, tumor size, grade, hormone receptors, and year of diagnosis). Grey curves: pointwise 95% confidence intervals of the *H_t_* hazard ratio at each percentage node. (b) Differences between successive hazard ratios obtained from graph (a), as a function of the percentage cutoff. The horizontal line is the mean of the differences *H_t_ – H_t–1_*.

Figure 1a represents the mortality hazard ratios comparing more vs. less nodal involvement as a function of the percentage of involved nodes. Applying the formalism of time series analyses, using the subscript *t* to denote the percentage of involved nodes as ordered observation and *H_t_* to designate the mortality hazard ratio at the percentage nodal involvement *t*, the hazard ratios were modeled as:

* H_t_ - H_t-1_ = μ + a_t_* (1)

where *μ* is a constant and a_t_ is a Gaussian random error with mean 0 and finite variance (white noise)

The constant *μ* was estimated as 0.020 (corresponding to a 2% increment in the mortality hazard ratio per 1% increase in the percentage of involved nodes). The variance of *a_t_* was estimated as 0.004. Though Figure 1a suggested some possible change points and the variance appeared to increase with increasing *t*, the hypothesis of a_t_ as white noise was nevertheless considered acceptable by the "portmanteau" test (P=0.205 for successive observations, i.e. the *a_t_* did not differ significantly from 0). Figure 1b shows the corresponding graphical display of the (*H_t_ - H*_t-1_)'s.

## Discussion

A model where *H_t_ - H_t-1_* is constant plus an error term denotes a random-walk with drift [9]. It is an integrated process in the sense of a recursive summation. The model can be rewritten as:

 *H_t_ = μ + a_t_ + (H_t-1_)* (2)

In other words, the mortality *H_t_* observed at the nodal percentage t is the sum of the preceding mortality risk *H_t-1_*, incremented by *μ* and *a*_t_*.* *H_t-1_* in its turn is the preceding mortality risk *H_t-2_*, incremented by *μ *and *a_t-1_*, *H_t-1_ = μ + a_t-1_ + (H_t-2_)*, and so on. The difference *H_t_ - H_t-1_* is stationary (Figure 1b), but the resulting mortality pattern is a summation of all previous risk increments with consequently an increasing drift (Figure 1a).

The model has limitations. The percentage of involved nodes is bounded, whereas random-walk and white noise assume an unlimited range. The percentages of involved nodes and the hazard ratios were computed on a population sample, the percentages and hazard ratios do not represent the individual risk. The time scale is unknown, changes in successive nodal involvements are not necessarily at a constant rate.

Despite the limitations, we nevertheless believe that the present analysis provides a novel insight into the course of breast cancer disease. Using the nodal percentage as a surrogate to time allows highlighting a simple fact: the axillary lymph node involvement observed at the moment of surgery did not occur overnight but represents a snapshot. The disease might have evolved for several years before diagnosis and might continue to further evolve after surgery. Equation (2) can be rewritten to explicitly show the cumulative relation:

 *H_t_ = μ∙t + H_0_ + (a_1_ + a_2_ + ... + a_t_)* (3)

where *H_0_* represents the risk of death at percentage 0. That is to say, the risk of death *H_t_* at percentage *t* depends on three terms. The first two terms correspond to the common linear relationship of the risk of death according to a disease-specific constant μ multiplied by the percentage of involved nodes and according to a baseline risk when no lymph nodes are involved. The last term, *(a_1_ + a_2_ + ... + a_t_)*, represents the sum of accumulated stochastic effects that occurred at all previous percentage points.

The constant *μ* might be considered an expression of disease intensity. A higher *μ* would imply a more aggressive disease, in which each % of nodal involvement and/or diagnostic delay incur a higher risk of death, and, conversely, a lower μ would imply a less severe disease. Recent researches in breast cancer molecular signatures suggest that indeed the prognostic impact of nodal ratios might be modulated by different gene expression profiles [10]. The stochastic a_t_ terms might reflect the effect of accumulated mutations. The model might potentially link with recent measures of tumor mutational burden [11].

Recently a Canadian team has modeled the SEER breast cancer data where the mortality by risk group could be explained by tumor dormancy, each characterized by a single stochastic rate from dormant to active [12]. Stochastic modeling of survival curves assigned the survival differences to differences in a tumor reactivation factor (α) and a multiplier (C). The team also reported a progression model in a drug simulation scenario, in which they found an apparent "delay in progression" due to reducing the number of women at risk of dying at time 0 but, otherwise, without any change of the underlying tumor biology, tumor growth had not been slowed [13]. Though our approach and model differ, we rejoin on the stochastic process.

Time underlies all biological processes. To our knowledge, the present study is the first ever to naturally apply time series formalism to lymph node count data. Time is not a requirement to apply time series analysis. The study used autocorrelation. "An autocorrelation is simply a correlation between two random variables Xt and Xs that are both part of the same time series (or other stochastic process, e.g. a spatial random field)" [14].

## Conclusions

Using the percentage of involved axillary lymph nodes, the effect of tumor burden on the risk of death in node-positive breast cancer could be concisely expressed as a random walk difference model, providing an in silico dynamic view of the process. In line with others, we will explore the hypothesis that effective treatment that changes the nature of the disease would reduce the *μ* and reduce the cumulative effect of the *a_t_* terms of the model.
